# CDC45 promotes the stemness and metastasis in lung adenocarcinoma by affecting the cell cycle

**DOI:** 10.1186/s12967-024-05038-5

**Published:** 2024-04-08

**Authors:** Yafeng Liu, Tao Han, Zhi Xu, Jing Wu, Jiawei Zhou, Jianqiang Guo, Rui Miao, Yingru Xing, Deyong Ge, Ying Bai, Dong Hu

**Affiliations:** 1 School of Medicine, Anhui University of Science and Technology, Chongren Building, No 168, Taifeng St, Huainan, 232001 People’s Republic of China; 2 Anhui Province Engineering Laboratory of Occupational Health and Safety, Anhui University of Science and Technology, Huainan, People’s Republic of China; 3 Key Laboratory of Industrial Dust Prevention and Control & Occupational Safety and Health of the Ministry of Education, Anhui University of Science and Technology, Huainan, People’s Republic of China; 4 Department of Clinical Laboratory, Anhui Zhongke Gengjiu Hospital, Hefei, People’s Republic of China; 5 Joint Research Center for Occupational Medicine and Health of IHM, School of Medicine, Anhui University of Science and Technology, Huainan, People’s Republic of China

**Keywords:** Lung adenocarcinoma, Stemness, Metastasis, Cell cycle, CDC45

## Abstract

**Objective:**

This study aimed to assess the functions of cell division cycle protein 45 (CDC45) in Non-small cell lung cancer (NSCLC) cancer and its effects on stemness and metastasis.

**Methods:**

Firstly, differentially expressed genes related to lung cancer metastasis and stemness were screened by differential analysis and lasso regression. Then, in vitro, experiments such as colony formation assay, scratch assay, and transwell assay were conducted to evaluate the impact of CDC45 knockdown on the proliferation and migration abilities of lung cancer cells. Western blotting was used to measure the expression levels of related proteins and investigate the regulation of CDC45 on the cell cycle. Finally, in vivo model with subcutaneous injection of lung cancer cells was performed to verify the effect of CDC45 on tumor growth.

**Results:**

This study identified CDC45 as a key gene potentially influencing tumor stemness and lymph node metastasis. Knockdown of CDC45 not only suppressed the proliferation and migration abilities of lung cancer cells but also caused cell cycle arrest at the G2/M phase. Further analysis revealed a negative correlation between CDC45 and cell cycle-related proteins, stemness-related markers, and tumor mutations. Mouse experiments confirmed that CDC45 knockdown inhibited tumor growth.

**Conclusion:**

As a novel regulator of stemness, CDC45 plays a role in regulating lung cancer cell proliferation, migration, and cell cycle. Therefore, CDC45 may serve as a potential target for lung cancer treatment and provide a reference for further mechanistic research and therapeutic development.

**Supplementary Information:**

The online version contains supplementary material available at 10.1186/s12967-024-05038-5.

## Introduction

Lung cancer is the leading cause of cancer-related deaths worldwide, posing a significant public health problem [1]. Non-small cell lung cancer (NSCLC) accounts for 80%-85% of lung cancer cases, with lung adenocarcinoma (LUAD) being the predominant subtype of NSCLC. Advanced LUAD has a poor prognosis, with high recurrence and metastasis rates, and a low 5-year survival rate [2].

Cancer stem cells (CSCs) have been identified as the primary driving force behind cancer recurrence. Increasing evidence supports the notion that poor prognosis in tumors is predominantly attributed to the acquisition of stemness properties by cancer cells [3, 4]. CSCs possess the ability of self-renewal [5, 6]^]^, which is the process by which CSCs divide to generate more stem cells [7, 8]. This process requires cell cycle control, and previous studies have indicated that the cell cycle is the primary regulatory factor in self-renewal [9]. The dynamic changes in gene expression are regulated by specific cell cycle proteins and cell cycle-dependent kinases (CDKs) that control cell cycle progression [10]. Studies have shown that silencing cell cycle proteins can regulate pluripotency factors, such as *OCT4*, *SOX2*, and *NANOG*, resulting in proteasomal degradation and a decline in stem cell pluripotency [11]. Although some studies have reported the self-renewal of stem cells [12], the mechanism between stemness and cell cycle remain unclear.

There is increasing evidence linking CSCs to tumor occurrence at metastatic sites and tumor recurrence after treatment [13]. The invasion and migration of tumor cells are also associated with the aggressive behavior of cancer [14]. Through epithelial-mesenchymal transition (EMT), epithelial cells lose their polarity, disengage from the basement membrane and other epithelial phenotypes, and acquire increased migration and invasive abilities, accompanied by extracellular matrix degradation and increased stromal phenotypes [15, 16]. Therefore, understanding the stem cell-like characteristics of cancer and the molecular mechanisms underlying metastasis is essential for improving the prognosis and recurrence of lung cancer.

CDC45, a critical regulator of the cell cycle, plays a vital role in the process of cell division. Elevated expression of CDC45 can induce DNA replication stress and abnormal cell cycle progression [17]. With further research on CDC45, it has been discovered that its expression level is closely related to disease progression in malignant squamous cell carcinoma, cervical cancer, prostate cancer, and lung cancer [18]. CDC45 is even involved in the proliferation, invasion, tumor angiogenesis, and formation of drug resistance in tumor cells [19–21]. However, the role of CDC45 in tumor stemness and lymph node metastasis has been rarely reported.

This study integrates multiple lung cancer-related datasets to identify differentially expressed genes (DEGs) in distinct stemness and lymph node metastasis states, and employs a series of bioinformatics methods to sort and analyze these DEGs. Ultimately, we validated that high expression of CDC45 promotes tumor stemness and lymph node metastasis in non-small cell lung cancer by regulating the cell cycle.

## Materials and methods

### Data collection

GSE35603 (Lung Cancer Tumor Stem-Like Cells, CD133( +), n = 3; Lung Cancer Parental Tumor Cell, CD133(−), n = 3), GSE166722 (tumor samples, n = 51), GSE68465(tumor samples, n = 442), GSE72094 (tumor samples, n = 398), and GSE31210 (tumor samples, n = 226) were downloaded from the Gene Expression Omnibus (GEO, http://www.ncbi.nlm.nih.gov/geo/). And, in these datasets we removed normal samples and tumor samples with no survival time. The mRNA expression levels and clinical information of TCGA-LUAD patients were downloaded from The Cancer Genome Atlas (TCGA). In addition, mutation data of LUAD patients were downloaded from the TCGA database.

### Differential gene analysis

From the GSE35603 and GSE166722 datasets, differentially expressed genes (DEGs) were accessed using the R package "limma" with criteria of |log2FC|≥ 1 and p < 0.05. Then, GO Biological Process enrichment analyses were performed on the DEGs. Furthermore, lasso analysis was conducted on the GSE166722 dataset to further screen for key genes. The expression of key genes and their relationship with pathologic conditions were observed in the TCGA patients, and univariate Cox analysis was performed to investigate the prognostic value of the key gene expression.

### Mutation frequency analysis

We obtained data of Lung Adenocarcinoma (TCGA, PanCancer Atlas) from the cbioportal database (https://www.cbioportal.org/) and explored the mutation frequencies of the 4 key genes. After that, the correlation between patients' TMB and the 4 key genes was further analyzed.

### GeneMANIA database

The GeneMANIA database is a bioinformatics prediction and analysis database based on gene function. We here analyze the potential interaction network and function of the key gene based on the GeneMANIA database.

### Cell culture and CDC45 knockdown

The A549 and H1299 lung adenocarcinoma cell lines were obtained from the Cell Bank of the Chinese Academy of Sciences(National Collection of Authenticated Cell Cultures,SCSP-503). The cells were cultured in DMEM medium (C1199550, Gibco) supplemented with 10% fetal bovine serum (Hyclone), 100 U/mL penicillin, 100 U/mL streptomycin, and mycoplasma removal agent at 37 °C and 5% CO2. si_CDC45 and negative control siRNA targeting CDC45 were designed and produced by Sigma Genomics. H1299 and A549 cells were seeded in a 6-well plate and allowed to adhere overnight. Transfect siRNA using Lipofectamine 2000 (11668019, Thermo Fisher Scientific Inc), after 6 h of transfection, the transfection medium was replaced with a normal culture medium. When the cells reached 80% confluency, they were harvested for passaging or further experimental testing. (Serial number: si_CDC45_NC, sense5′-UUC UCC GAA CGU GUC ACG UTT-3 antisense5′-ACG UGA CAC GUU CGG AGA ATT-3′. si-CDC45#1, S: GGAUCUCCUUUGAGUAUGATT AS: UCAUACUCAAAGGAGAUCCTT. si-CDC45#2, S: CGAGCAGUAUGAAUAUCAUTT AS: AUGAUAUUCAUACUGCUCGTT. si-CDC45#3, S: GGAGGAUGAAGAGCAUUCATT AS: UGAAUGCUCUUCAUCCUCCTT).

### Colony formation assay

Control and CDC45 siRNA-transduced A549 and H1299 cells were cultured in 6-well plates at a density of 3000 cells per well and incubated at 37 °C in a 5% CO2 environment. After 10 days, the cells were stained with 4% formaldehyde/0.005% crystal violet solution, and the colony formation was observed under an inverted microscope.

### Scratch assay and transwell migration assay

Control siRNA and si_CDC45 cells were seeded in 6-well plates to allow for the formation of a monolayer, and a manual scratch was made using a 200 μl pipette tip. Subsequently, the cells were washed with PBS and incubated in a serum-free medium, and photographs of the scratched areas were taken every 24 h using a phase-contrast microscope. Cell migration and invasion assays were performed using a 1 × 10^4 cell suspension in 200 μl of 1% BSA-containing medium in the upper chamber, followed by the addition of 600 μl of 10% FBS-containing medium to the lower chamber. After incubation for 24 h, the cells on the upper chamber were washed away, and the migrated or invaded cells in the lower chamber were fixed with 4% paraformaldehyde at room temperature for 20 min. Subsequently, the cells were stained with 0.01% crystal violet for 20 min and imaged under a microscope. The data presented herein are derived from at least three independent experiments.

To measure cell migration ability, a 24-well transwell chamber (3422, Corning,8.0um) was used. Serum-free medium was added to the upper chamber, while serum-containing medium was added to the lower chamber. Cells were seeded in the upper chamber at a density of 1 × 10^4^ cells per well and incubated at 37 °C in 5% CO2 for 24 h. The cells that migrated to the lower chamber were fixed and stained using methanol and 0.5% crystal violet, respectively. Images of each well were captured using an inverted microscope.

### Western blotting

Equal amounts of whole-cell extracts were separated by 10% SDS-PAGE and transferred onto PVDF membranes. The membranes were blocked in 5% skim milk and incubated with specific primary antibodies (E-cad, N-cad, Vimentin, CDK2, CDK4, NIFK, NAONG, and β-Actin; diluted 1:1000) overnight, followed by incubation with the appropriate secondary antibodies (diluted 1:10,000 Abclonal catlog:AS014). The immunoreactivity was visualized using a chemiluminescent detection kit (WBKLS0100, Millpore). The primary antibodies used for E-cad, N-cad, Vimentin, and Naong were purchased from CST (catlog:3195 T, 841175SF, 5741 T, 4903 T), CDK2 and CDK4 were purchased from PTG (catlog:10122–1-AP,11026–1-AP), NIFK and *β*-actin was purchased from Abclonal(catlog:A15595, AC026). All measurements were performed in triplicate.

### Flow cytometry for cell cycle detection

Cells were trypsinized and washed in cold PBS, then fixed in 75% ethanol overnight. Cells were stained with PI/RNase staining buffer and incubated at 4 °C for 60 min. Cell cycle distribution was analyzed using a FACS Calibur flow cytometer.

### Subcutaneous tumor formation

C57BL/6 mice (4 weeks old) were purchased from Henan Sczbio Biotech Co., Ltd. (Henan, China) and housed under specific pathogen-free conditions in a laminar airflow cabinet. For the subcutaneous LLC tumor model, 1 × 10^6 LA-4 cells (Group 1: sh_NC; Group 2: sh_CDC45) were injected subcutaneously into the right flank of each mouse (8/group). Mice were monitored regularly during the study, and tumor size was measured every 5 days using calipers (tumor volume calculation: length × width × width × π/6). After 6 weeks, mice were euthanized, and tumors were excised, weighed, and further analyzed.

### Statistical analysis

All measurements were performed in triplicate in three independent experiments, and quantitative data are presented as mean ± SEM. Differences between the two groups were compared using a two-tailed Student's t-test. The R package "pheatmap" was used to generate volcano plots to represent the results of variance analysis. LASSO regression (R packages “glmnet”) and Univariate Cox regression analysis (R packages “survival”) were used to identify candidate genes.The R package "clusterProfiler" was used for GO analysis. In all cases, p < 0.05 was considered statistically significant.

## Results

### Identification of genes associated with lung adenocarcinoma metastasis and stemness

To investigate the potential link between cell stemness levels and lymph node metastasis. We obtained CD133 + (Tumor Stem-Like Cells) and CD133- (Parental Tumor Cells) LUAD cells from the GSE166722 dataset, which is a LUAD cohort of early stage (stage I&II) patients with lymph node metastasis information, and the GSE35603 dataset.

**Fig. 1:**
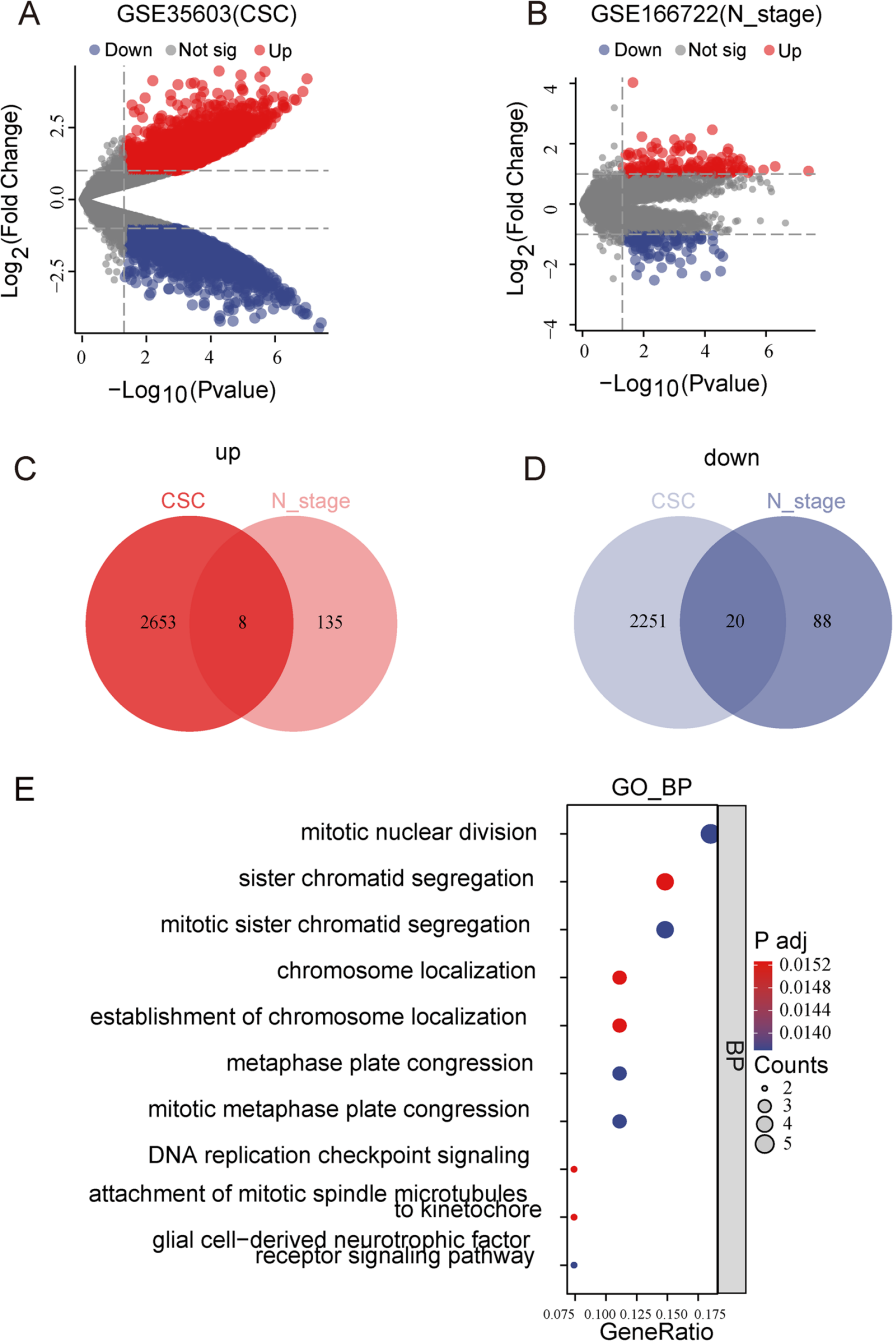
Identification of genes associated with metastasis and stemness in LUAD. **A** DEGs associated with stemness in LUAD from GSE35603. **B** DEGs associated with metastasis in LUAD from GSE166722. **C, D** Venn diagrams identifying genes associated with metastasis and stemness in LUAD. **E** Bubble plot showing GO functional enrichment analysis of co-expressed genes

We collected 2661regulated and 2371 downregulated stemness-associated DEGs in LUAD from the GSE35603 dataset (Fig. 1A). Furthermore, we collected 143 upregulated and 108 downregulated metastasis-associated DEGs in LUAD from the GSE166722 dataset (Fig. 1B). By integrating the differential analysis results from these two datasets, we obtained 8 overlapping upregulated genes (Fig. 1C) and 20 overlapping downregulated genes (Fig. 1D). Subsequently, the 28 overlapping genes were subjected to GO enrichment analysis, which revealed their enrichment in biological processes related to cell proliferation, such as nuclear division, sister chromatid segregation, chromosome localization, midbody assembly during mitosis, DNA replication checkpoint signaling pathway, mitotic spindle attachment, and glial cell-derived neurotrophic factor receptor signaling pathway (Fig. 1E). Overall, we screened for genes associated with cell stemness that are important in promoting cell proliferation, and division.

### Selection of key genes using lasso analysis in GSE166722

**Fig. 2:**
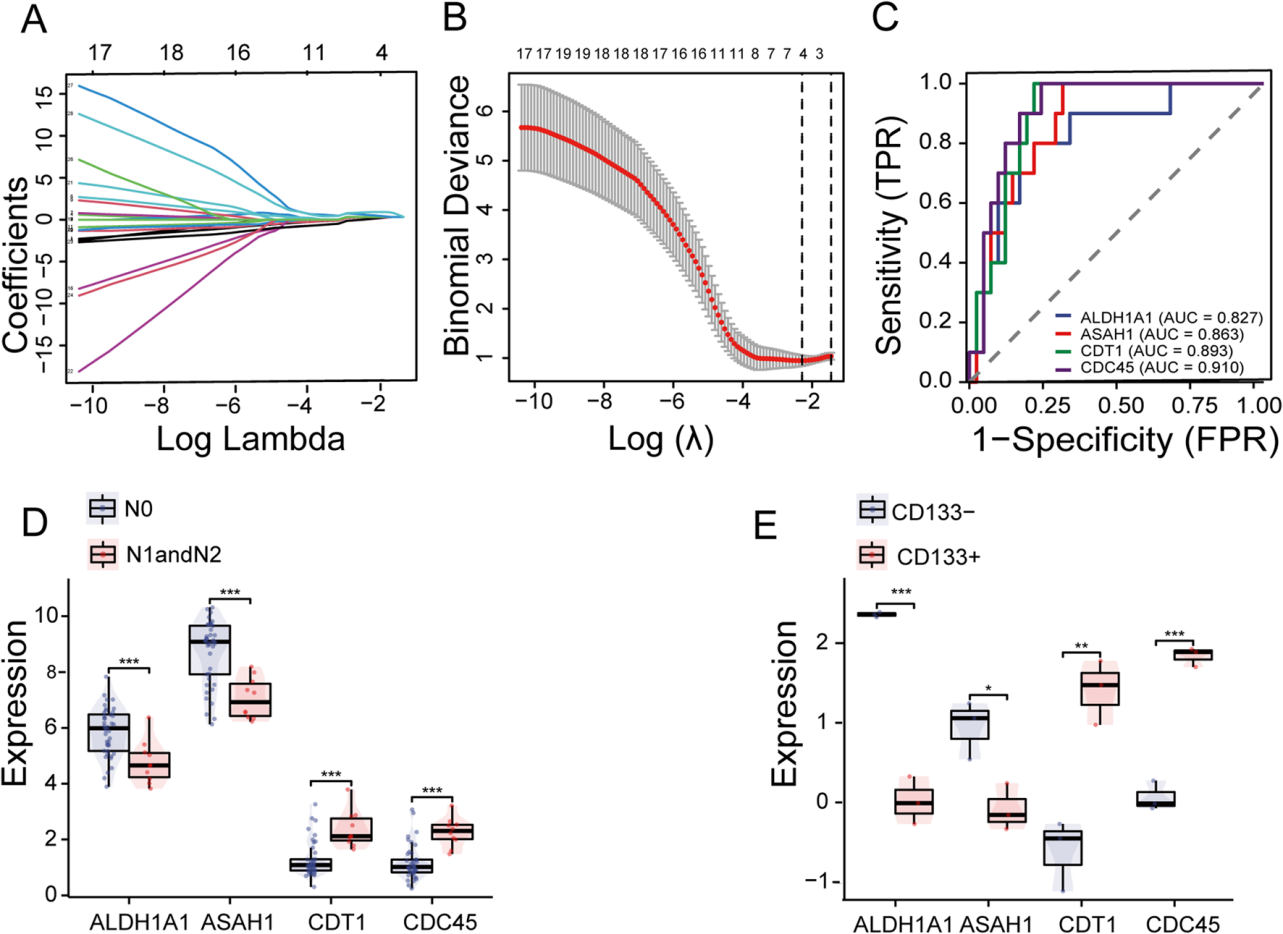
Identification of metastasis-related genes. **A** Determination of the optimal lambda value based on the minimum partial likelihood deviance. **B** LASSO coefficient profiles of candidate genes for the diagnostic model. **C** ROC curves of key genes for diagnosing lymph node metastasis. **D** Distribution of key genes in N0 and N1&N2. **E** Expression profiles of key genes in CD133 + and CD133- cells. ***p < 0.001

To select the most predictive feature genes for LUAD stemness and metastasis from the 28 candidate genes, Lasso regression analysis was performed on the GSE166722 dataset, resulting in the selection of 4 key genes associated with lymph node metastasis (Fig. 2A, B, C). Subsequently, the relationship between these 4 feature genes and lymph node metastasis and stemness was analyzed. The results showed that CDC45 and CDT1 were highly expressed in the lymph node metastasis group, while *ALDH1A1* and *ASAH1* were downregulated (Fig. 2D). Moreover, *CDC45* and *CDT1* were highly expressed in the CD133 + population, while *ALDH1A1* and *ASAH1* were downregulated in the CD133- population (Fig. 2E). These results suggest an important role for *CDC45* and *CDT1* in identifying high stemness cells and promoting lymph node metastasis.

### Correlation analysis between key genes and clinicopathological parameters in TCGA

**Fig. 3:**
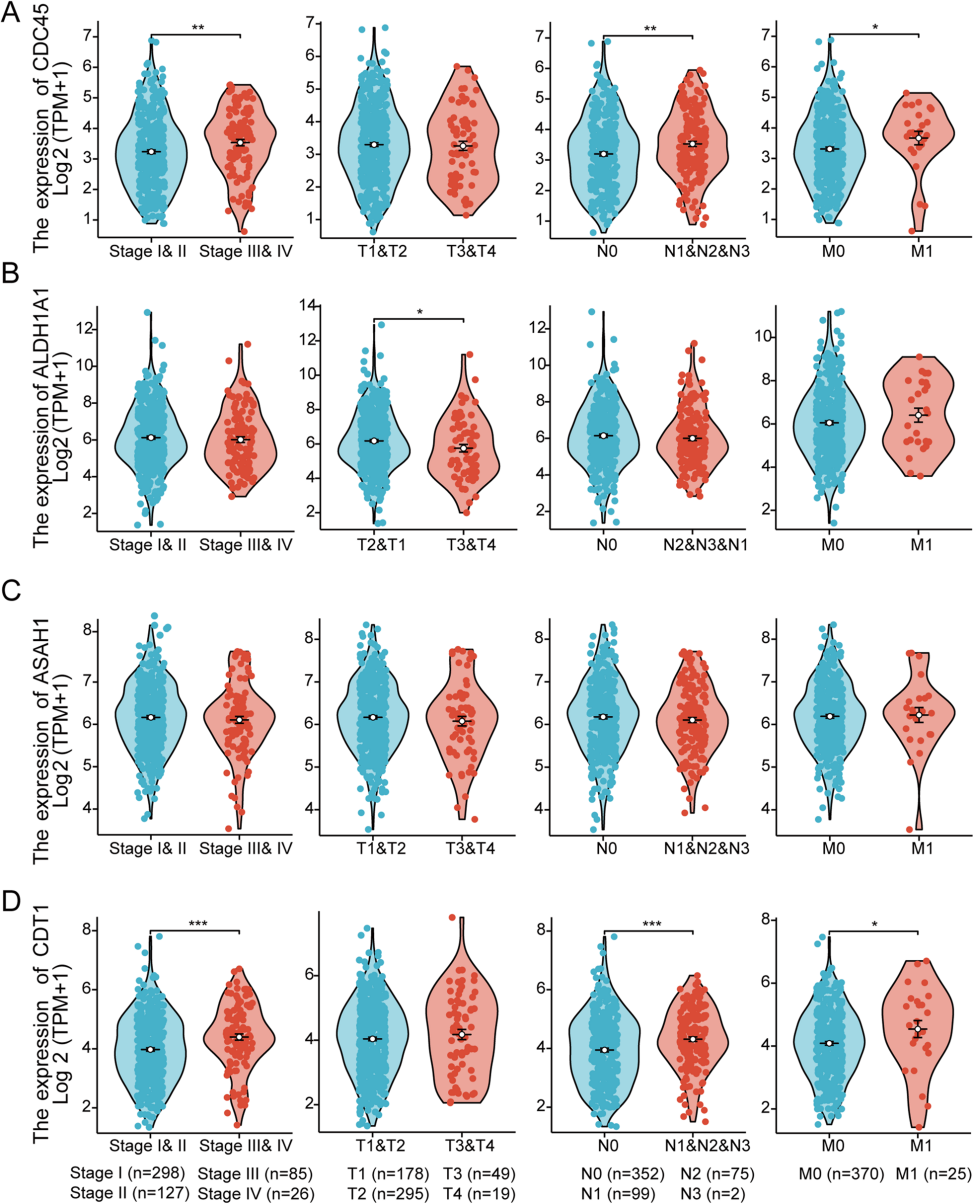
Association between key genes and clinical pathological staging in TCGA. **A** *ALDH1A1*, **B** *ASAH1*, **C** *CDC45*, and **D** *CDT1* expression differences in different populations and tumor stages

To explore the potential role and prognostic value of key genes in tumor progression, the relationship between the 4 key genes and clinical pathologic staging was analyzed. The results showed that the expression of *ALDH1A1* was not significantly different in terms of age, gender, tumor staging, lymph node metastasis, and distant metastasis (*P* > 0.05), but it was associated with tumor diameter (P < 0.05) (Fig. 3A). The expression of *ASAH1* showed no significant differences in age, gender, tumor staging, tumor diameter, lymph node metastasis, and distant metastasis (Fig. 3B). The expression of *CDC45* was associated with age, gender, tumor staging, and lymph node metastasis (*P* < 0.05), but showed no significant differences in tumor diameter and distant metastasis (*P* > 0.05) (Fig. 3C). The expression of *CDT1* was associated with age, gender, tumor staging, and lymph node metastasis (*P* < 0.05), but showed no significant differences in tumor diameter and distant metastasis (*P* > 0.05) (Fig. 3D). Overall, the consistency of these results suggests an association between *CDT1* and *CDC45* and the progression of the LUAD malignant clinical phenotype.

### Positive correlation of key genes with stemness and impact on prognosis of lung adenocarcinoma patients

**Fig. 4:**
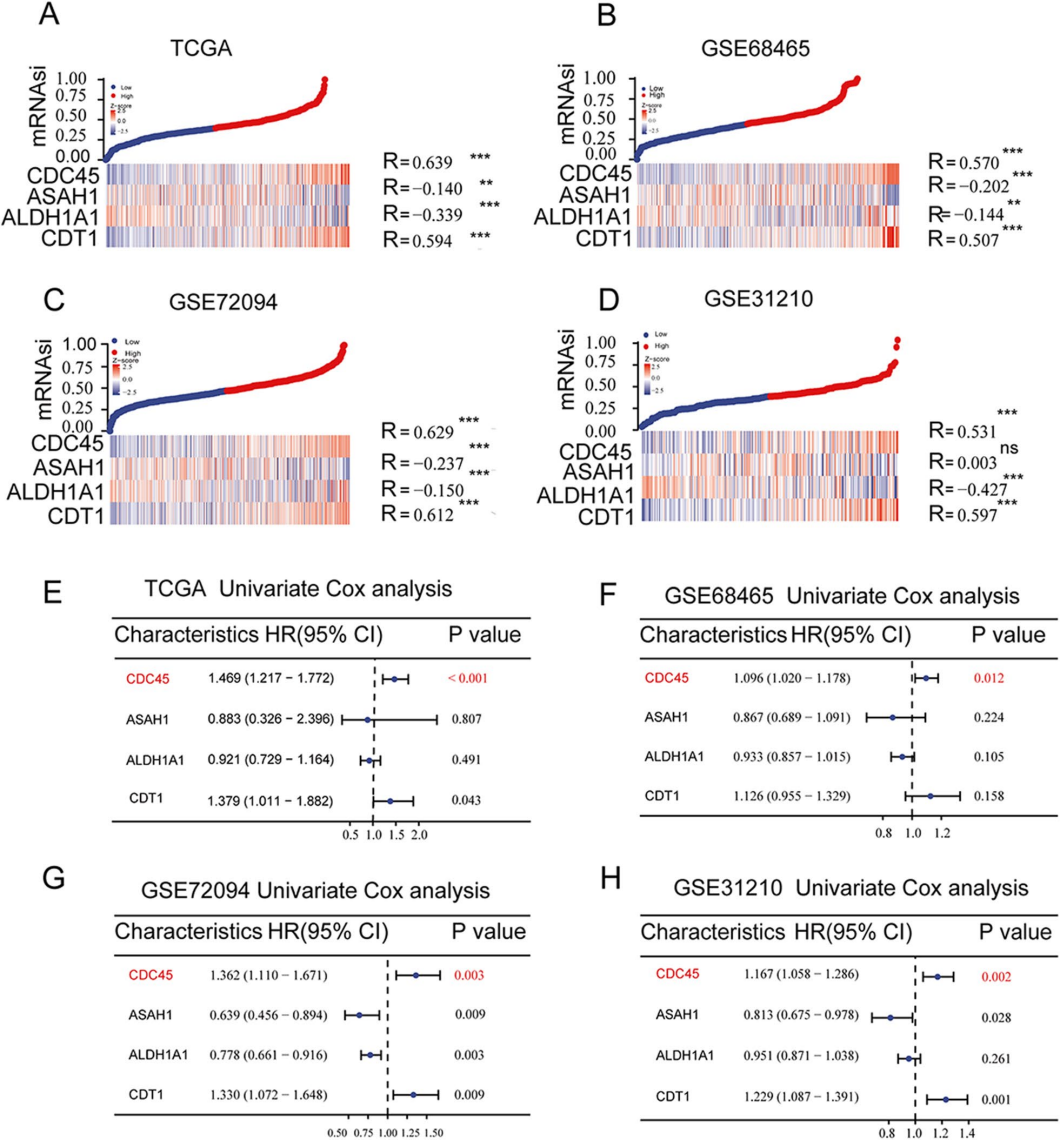
Correlation between key genes and stemness and prognosis. **A**–**D** Heatmaps showing the correlation between key genes and stemness in TCGA, GSE68465, GSE72094, and GSE31210 datasets. **E**–**H** Forest plots of univariate regression analysis of key genes in TCGA, GSE68465, GSE72094, and GSE31210 datasets

The correlation between key genes and stemness was further investigated, by analyzing data from the TCGA, GSE68465, GSE72094, and GSE31210 datasets. The results showed that *CDC45* and *CDT1* were positively correlated with stemness in all four datasets (R > 0.5, *P* < 0.01), while *ASAH1* and *ALDH1A1* were negatively correlated with stemness (R < − 0.1, *P* < 0.01) (Fig. 4A–D). Furthermore, the impact of the four key genes on survival analysis was evaluated. Univariate Cox regression analysis was performed based on the gene expression in the aforementioned four datasets. The results showed that *CDC45* and *CDT1* were positively associated with death risk rate (HR > 1, *P* < 0.05), while *ASAH1* and *ALDH1A1* were not statistically significant (*P* > 0.05) (Fig. 4E). This result implies that *CDC45* may play an important role in contributing to the low survival of LUAD patients.

### Functional analysis of key gene *CDC45*

**Fig. 5:**
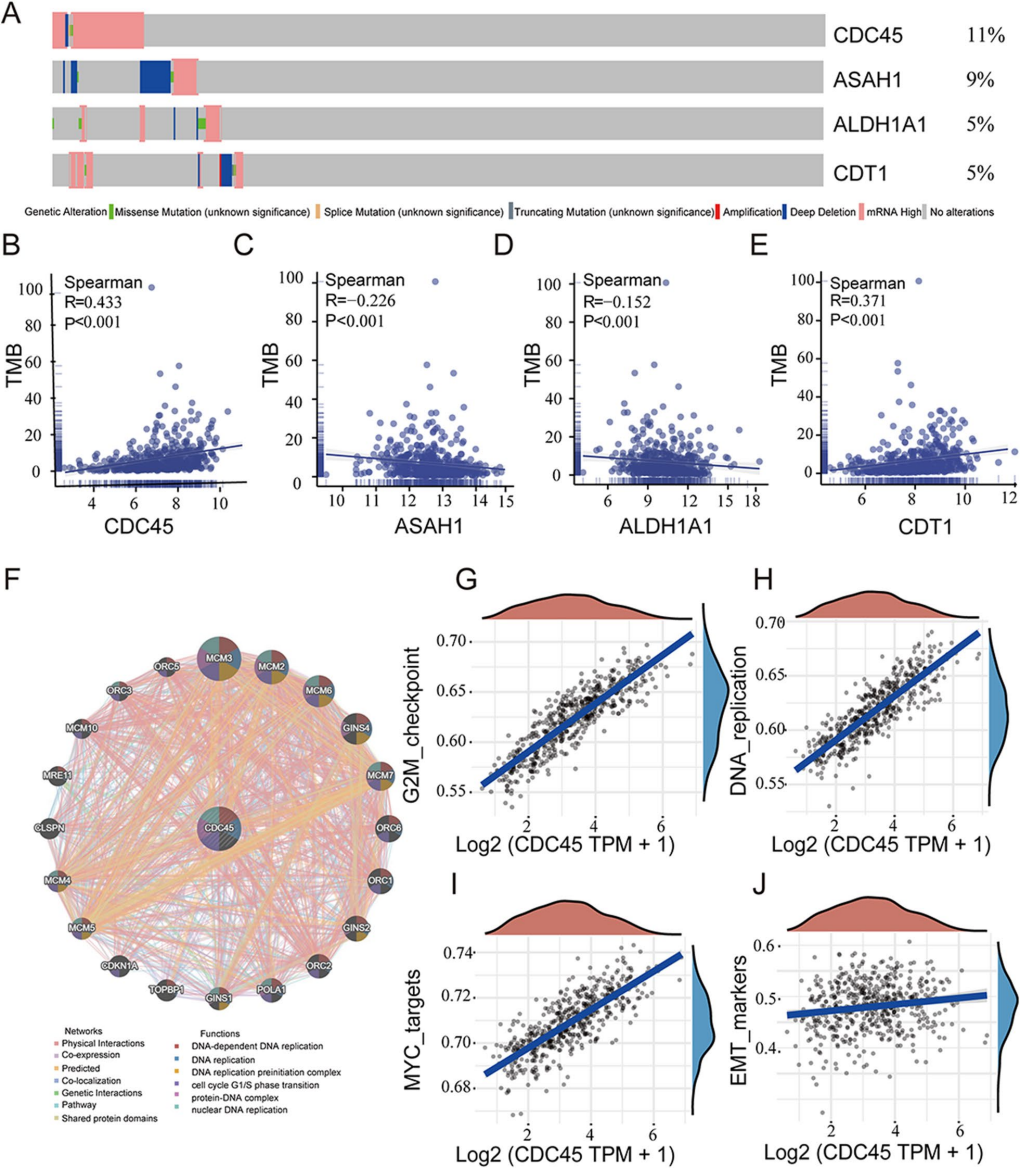
Relationship between key genes and mutations and carcinogenesis pathways. (**A**) Mutation frequency visualization of key genes using the cBioPortal database. **B–E** Scatter plots showing the correlation between genes and tumor mutation burden (TMB). **F** Network of CDC45 and co-expressed genes based on the genemania database. **G**–**J** Correlation between CDC45 and stemness and metastasis-related pathways

To further elucidate the involvement of the 4 genes in tumor mutations, the cBioPortal database was used to visualize the mutation rates of the key genes. It was found that *CDC45* had the highest mutation rate at 11% among the 4 feature genes (Fig. 5A). Furthermore, since tumor mutational burden (TMB) is positively correlated with oncogenic mutations and immune therapy response, the correlation between TMB and the 4 key genes was analyzed [22]. The results showed a significant positive correlation between *CDC45* and TMB (R = 0.433, *P* < 0.001) (Fig. 5B), a negative correlation between *ASAH1* and TMB (R = − 0.226, *P* < 0.001) (Fig. 5C), a negative correlation between *ALDH1A1* and TMB (R = − 0.152, *P* < 0.001) (Fig. 5D), and a positive correlation between *CDT1* and TMB (R = 0.371, *P* < 0.001) (Fig. 5E). Combined with the univariate survival analysis, it was confirmed that *CDC45* was positively correlated with stemness and metastasis in LUAD and influenced the prognosis of LUAD.

To further study the potential protein interactions of *CDC45*, potential interactions were retrieved using the Gene MANIA database, and a protein–protein interaction (PPI) network was constructed. The functional enrichment analysis showed enrichment in functions related to DNA replication, cell cycle G1/S transition, nuclear DNA replication, and others (Fig. 5F). Subsequently, the correlation of *CDC45* with the G2/M checkpoint cell cycle signaling pathway, DNA replication signaling pathway, cell proliferation-related signaling pathways, and EMT was analyzed, and positive correlations were observed (Fig. 5G, H, I and J). In conclusion, the link that exists between *CDC45* and mutational load explains in part why *CDC45* is associated with poorer survival outcomes.

### CDC45 Knockdown inhibits cell proliferation and migration

**Fig. 6:**
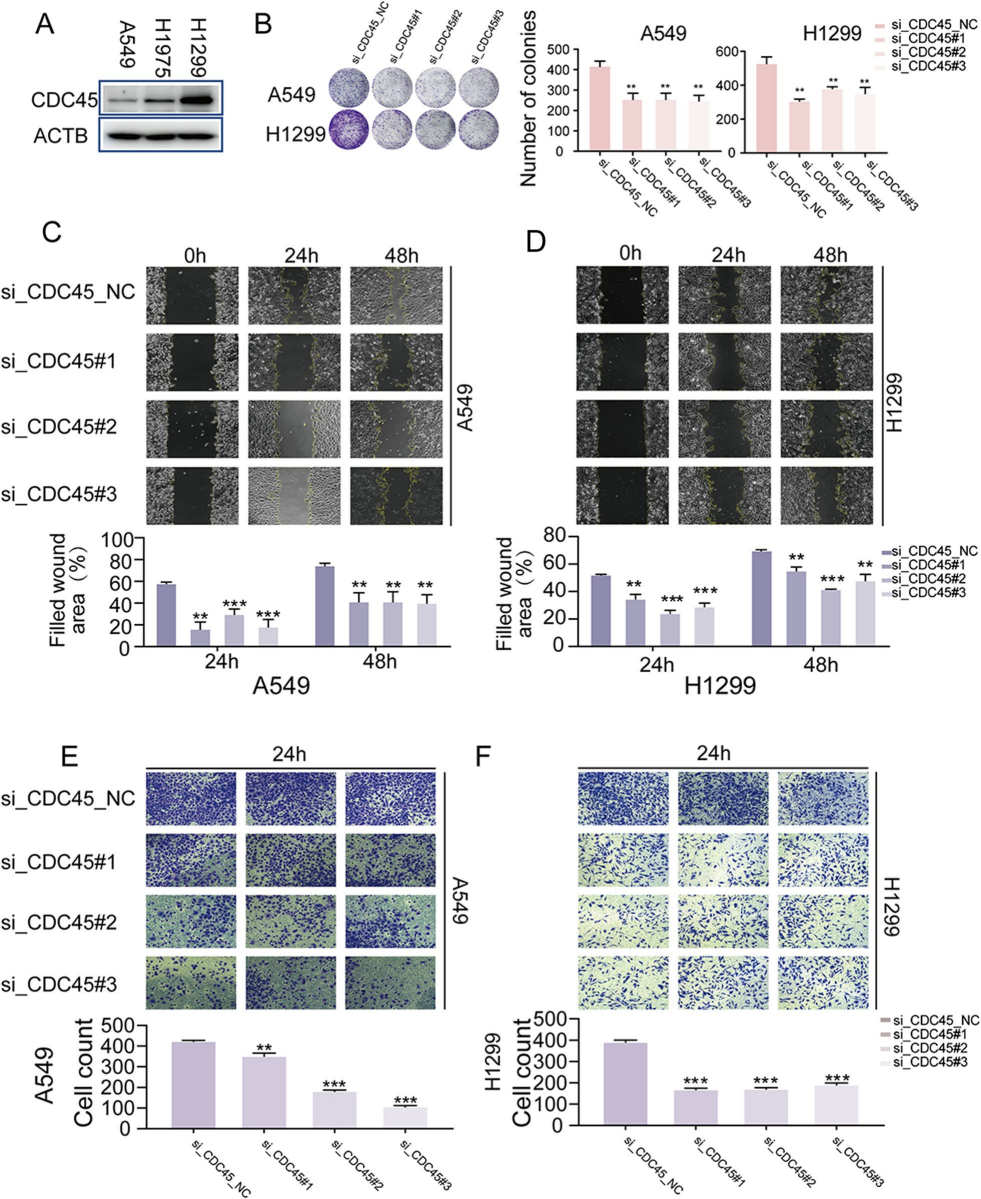
Knockdown of CDC45 reduce cell proliferation and migration. **A** WB validation of CDC45 expression in three cell lines. **B** Colony formation assay demonstrating the inhibition of cell proliferation by CDC45 knockdown. **C–D** Scratch assay showing the inhibition of cell migration by CDC45 knockdown. **E**, **F** Transwell assay validating the inhibition of cell migration by CDC45 knockdown

WB analysis was performed to examine the protein expression level of CDC45 in the three cell lines. The results showed that CDC45 protein expression was highest in H1299, followed by H1975, while A549 exhibited the lowest expression level (Fig. 6A). Additionally, considering the heterogeneity of cancer cells, we performed immunofluorescence staining on A549 and H1299 cells. The detection results revealed that the majority of cells in both A549 and H1299 cell lines expressed CDC45 (Additional file 1: Fig. S1). A549 and H1299 cells were selected for further experiments. To determine the function of CDC45 in A549 and H1299 cells, CDC45-specific siRNA was transfected to knock down CDC45 expression. Initially, we conducted WB experiments to assess the knockdown efficiency of CDC45. The results indicated that CDC45 was successfully knocked down in A549 and H1299 cell lines (Additional file 1: Fig. S2). Compared to the control group, the tumor cell colonies formed after the CDC45 knockdown were smaller and the number of colonies decreased (Fig. 6B). Scratch assays and transwell assays were performed to evaluate cell migration ability. The scratch assay results showed that CDC45 knockdown in H1299 and A549 cells reduced their migration ability compared to cells transfected with control siRNA (Fig. 6C, D). The transwell assay results demonstrated that CDC45 knockdown inhibited cell migration, as the number of migrated cells was reduced compared to the control siRNA group (Fig. 6E, F). These results indicate that CDC45 knockdown inhibits proliferation and migration in H1299 and A549 cells.

### CDC45 Knockdown inhibits EMT in LUAD Cells

**Fig. 7:**
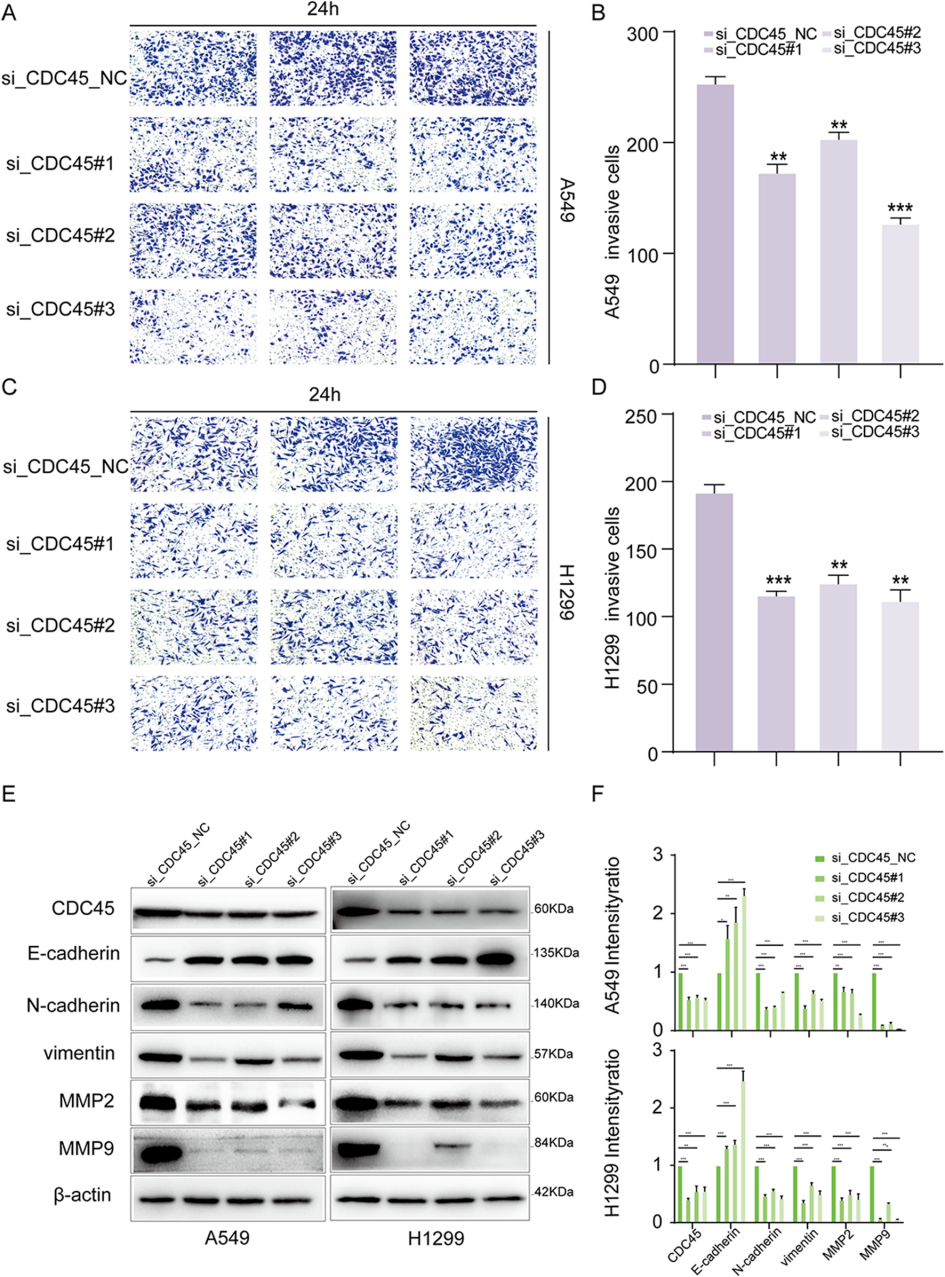
Knockdown of CDC45 inhibits EMT in H1299 and A549 cells. **A**, **B** Representative pictures and quantitative analysis of the inhibitory effect of CDC45 knock-down on cell invasion in A549 cells. **C**, **D** In H1299 cells, the representative pictures and quantitative analysis of the inhibitory effect of CDC45 knock-down on cell invasion. **E**, **F** Western blot assay, after knockdown CDC45, expression levels in Vimentin, E-cadherin, and N-cadherin in A549 and H1299 cells decreased, while knockdown CDC45 reduced levels of MMP2 and MMP9, with the reference protein*β*-actin used as an internal control

To investigate whether invasion relies on CDC45, we first performed transwell invasion experiments. The results demonstrated that in A549 cells, knocking down CDC45 inhibited cell invasion (Fig. 7A, B). Similar effects were observed in H1299 cells (Fig. 7C, D). To further clarify the mechanisms underlying the enhancement of cell proliferation, migration, and invasion by high CDC45 expression, we analyzed the protein expression of EMT markers closely associated with tumor metastasis in A549 and H1299 cells by WB analysis. The protein imprinting analysis showed that the knockdown of CDC45 in A549 cells increased the expression of E-cadherin, while the expression of Vimentin and N-cadherin decreased compared to the control group. Additionally, the expression levels of MMP2 and MMP9 were decreased in the CDC45 knockdown group compared to the control group. Similar results were observed in H1299 cells (Fig. 7E, F).

### Cell cycle blockade by CDC45 knockdown

**Fig. 8:**
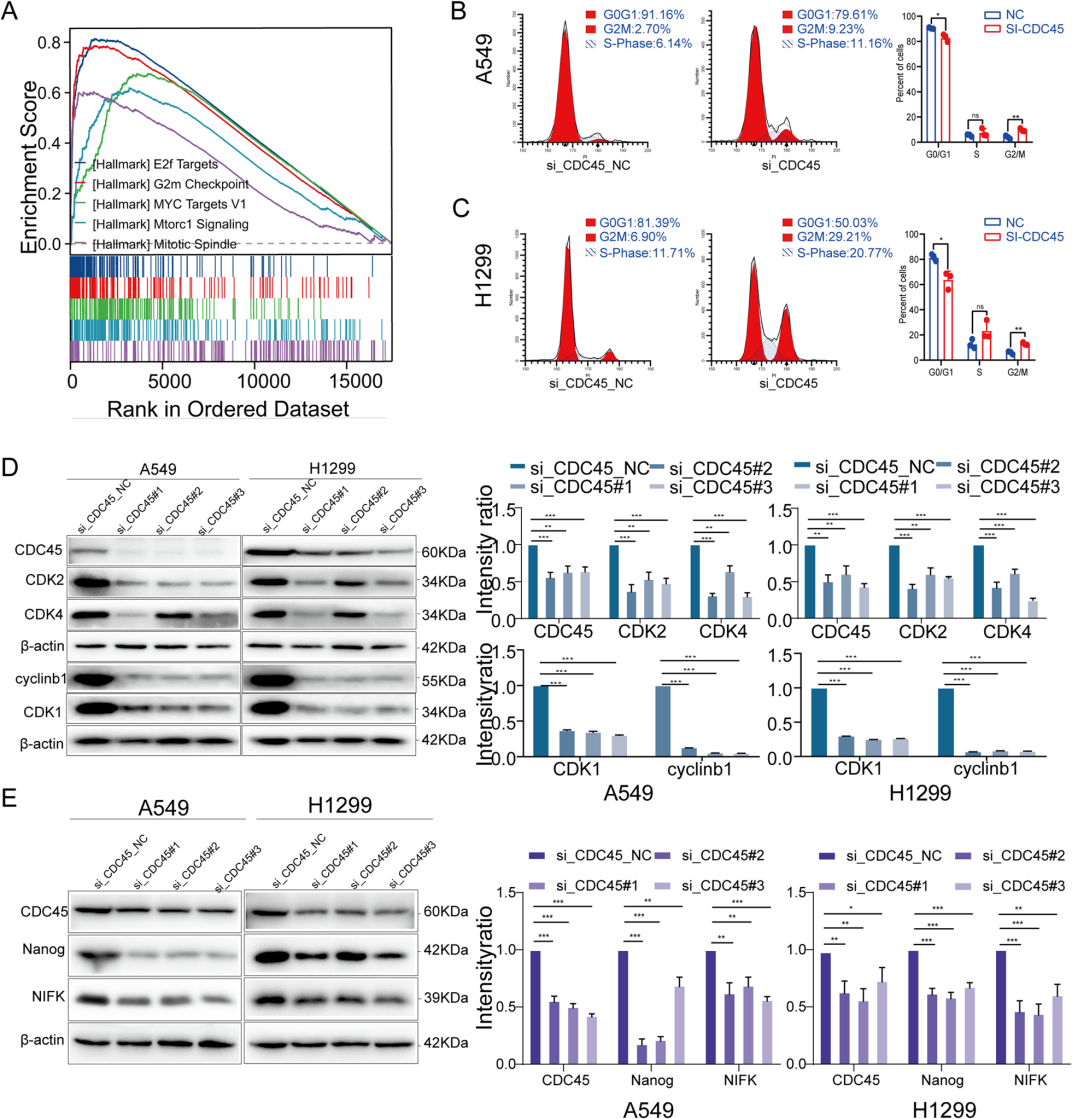
Knockdown of CDC45 inhibits cell cycle progression. **A** GSEA pathway enrichment analysis. **B**, **C** Flow cytometry analysis of cell cycle distribution. **D** Protein expression of CDK2, CDK4, CDK1 and cyclin B1 in si-CDC45 transfected A549 and H1299 cells. **E** Protein expression of Nanog and NIFK in si-CDC45 transfected A549 and H1299 cells

Cell cycle dysregulation is a hallmark of cancer cells Therefore, inducing cell cycle arrest may be an effective strategy for controlling abnormal cancer cell proliferation. Initially, GSE analysis revealed that CDC45 overexpression was mainly enriched in cell cycle, cell cycle processes, DNA replication, and DNA metabolic processes (Fig. 8A). Subsequently, flow cytometry analysis was performed after the CDC45 knockdown, and the results showed that the knockdown of CDC45 led to cell cycle arrest at the G2-M phase (Fig. 8B, C). Cyclin-dependent kinases (CDKs) are a group of serine/threonine protein kinases that drive the cell cycle through their chemical effects on serine/threonine proteins. CDKs play an important role in cell cycle regulation and are involved in driving the cell cycle. Therefore, we also examined the effects of CDC45 on CDK2 and CDK4 through WB analysis. The results showed that the knockdown of CDC45 reduced the expression levels of CDK2 and CDK4 (Fig. 8D). The expression of stemness markers was analyzed through WB. The protein imprinting analysis showed that the knockdown of CDC45 in H1299 cells resulted in a decrease in Nanog and NIFK protein expression compared to the control group (Fig. 8E). These results suggest a link between CDC45 and cell stemness and cell cycle.

### Inhibition of subcutaneous tumor growth in mice by CDC45 knockdown

**Fig. 9:**
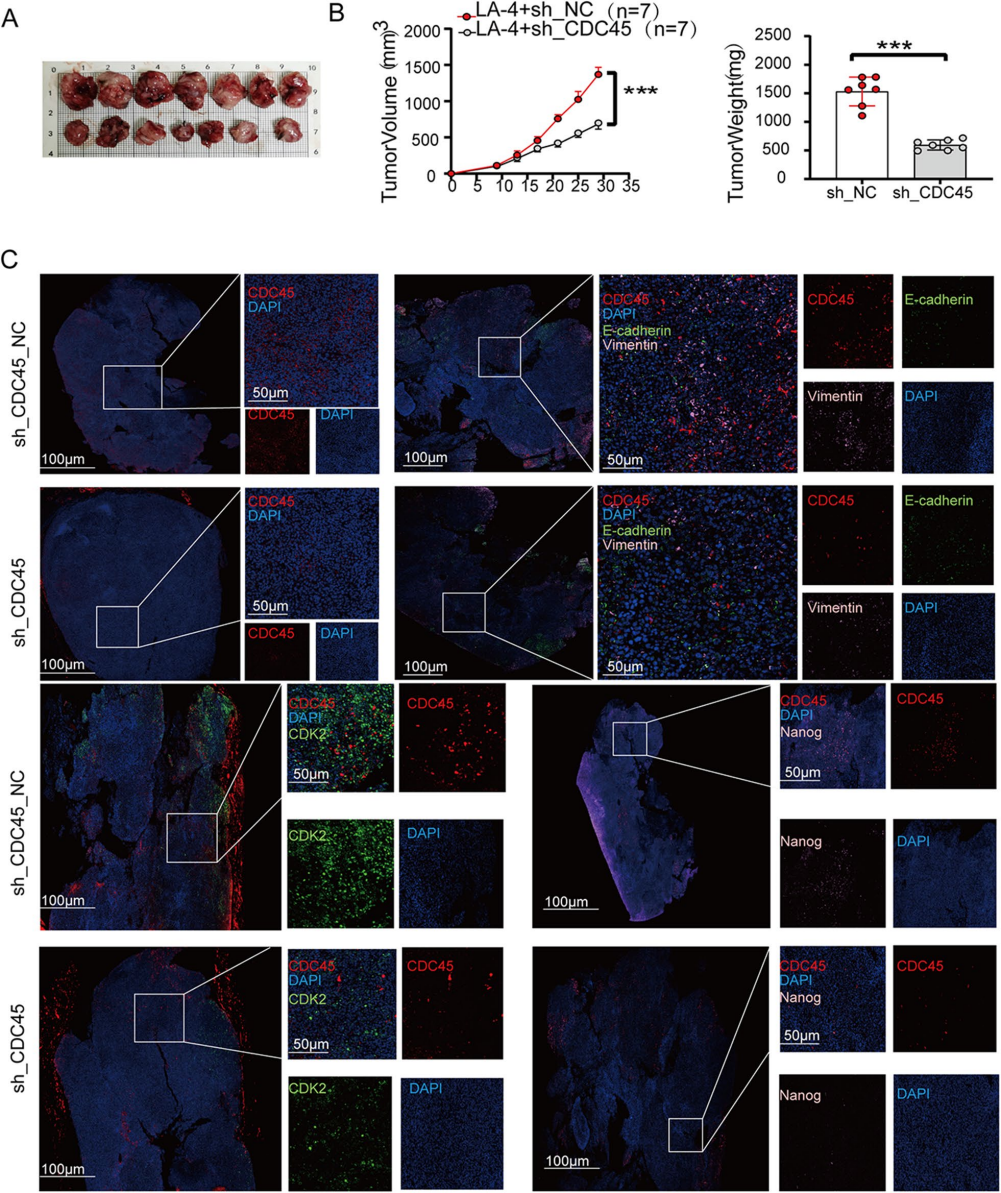
Animal experiment. **A** Photos of tumor sizes. **B** Statistical analysis of subcutaneous tumor sizes and body weight. **C** Immunofluorescence analysis of tumor tissues

To further investigate the role of CDC45 and validate it as a potential therapeutic target in vivo, C57BL/6 mice were injected with LA-4 lung cancer cells transduced with lentiviral particles containing CDC45-RNAi (Additional file 1: Fig. S3). Compared to the control group, the mice injected with CDC45-RNAi lentivirus showed smaller tumor sizes (Fig. 9A, B). Immunofluorescence staining showed that the immunofluorescent signal of tumors was significantly reduced in mice transduced with CDC45-RNAi lentivirus compared to the control group (Fig. 9C). At the same time, we quantitatively analyzed the immunofluorescence results (Additional file 1: Fig. S4). Therefore, the knockdown of CDC45 inhibited subcutaneous tumor growth in mice.

## Discussion

In our study, we identified four key genes (ALDH1A1, ASAH1, CDT1, and CDC45) that affect tumor cell stemness and lymph node metastasis. ALDH1A1 serves as a stem cell marker in various cancers and participates in the maintenance of cancer stem cells [23, 24]. Over-expression of ALDH1A1 is associated with poor prognosis in many cancers such as ovarian, gastric, breast, and colorectal cancers [25–28]. However, in lung cancer, the research on ALDH1A1 exhibits two extreme phenomena, particularly in ADC cases, where the restoration of ALDH1A1 expression significantly inhibits the growth of some lung cancer cell lines [29]. This finding supports the negative correlation between ALDH1A1 expression and stemness and metastasis in our study (Fig. 2D, E). In addition, in vitro experiments showed that reducing ALDH1A1 weakens the growth and migration of some lung cancer cell lines, indicating its carcinogenic effects [30]. The discrepancy between these two conclusions may be attributed to tumor cell heterogeneity [31], as different types of lung cancer cells may have distinct regulatory mechanisms for ALDH1A1 expression and function. ASAH1 plays an important role in regulating ceramide metabolism and tumor pathogenesis [31]. It can promote the proliferation of cancer cells and enhance the formation of tumor [32, 33]. However, according to our analysis of TCGA-LUAD data, no significant differences were observed between ASAH1 and histological grade, tumor size, lymph node metastasis, and distant metastasis (Fig. 3C). CDT1, a core regulator of DNA replication initiation, plays a vital role in cell cycle progression and DNA damage response [34]. In various tumors, including breast, lung, and lymphoma, high expression of CDT1 is associated with increased malignancy and decreased survival rates [35–37]. However, in our study, although CDT1 expression showed significant differences in tumor stage and lymph node metastasis, there were no expression differences in tumor size and distant metastasis (Fig. 3D). Furthermore, compared to the other three genes, the mutation frequency of CDT1 was lower (Fig. 5A).

CDCC45 is one of the proteins essential for the initiation and elongation of DNA replication and is necessary for regulating DNA replication [38]. CDC45 forms a "supercomplex" with the Chromosome Maintenance Complex (MCM) and Go-Ichi-Ni-San (GINS), which is the core of eukaryotic replication and has been demonstrated to have helicase activity [39]. It binds to DNA molecules and unwinds double-stranded DNA, forming replication fork structures during the entire DNA replication process. Moreover, previous studies have identified CDC45 as a proliferation-associated antigen and have shown its close association with the progression of various cancers, including cervical cancer [40], colorectal cancer [20], acute myeloid leukemia [41]^]^, and ovarian cancer [42].

CDC45 affects the normal progress of cell cycle by participating in the initiation of DNA replication and maintaining S-phase progress [43]. In our study, we verified the expression of cyclin-dependent kinases (CDK2 and CDK4) by WB analysis. The results showed that the knock-out of CDC45 reduced the expression level of CDK2 and CDK4 compared with the control group. In addition, flow cytometry analysis showed that knocking out CDC45 led to cell cycle arrest in G2/M phase. The reason for this phenomenon may be that CDC45 has been found to interact with cell cycle checkpoint proteins such as CHK1 and CHK2 and promote their activation. Cell cycle checkpoint is responsible for monitoring and controlling the repair of cell DNA damage and the coordination of cell cycle process at different stages of cell cycle. The regulation of CDC45 affects the activity of checkpoints, thus preventing the cell cycle from proceeding under DNA damage or other abnormal conditions [44].

There are many direct interactions between cell cycle regulators and the stemness pathway [45, 46]. Therapies targeting tumor cell stemness should consider the impact on the cell cycle to more effectively suppress tumor cell proliferation and metastasis. In this study, we demonstrated the downregulation of stemness markers (such as Nanog and NIFK) at the protein level in lung cancer cell lines A549 and H1299 upon CDC45 knockdown, indicating that the high expression of CDC45 in lung cancer cell lines is associated with increased stemness marker expression. Furthermore, Sun et al. found that CDC45 was upregulated in papillary thyroid carcinoma (PTC) and promoted the proliferation of cancer cells in vitro and tumor growth in vivo. Knockdown of CDC45 using siRNA led to cell cycle arrest and apoptosis inhibition [47, 48]. CDC45 may be involved in the regulation of processes related to epigenetic modification, such as chromatin remodeling and DNA modification. These processes play a key role in stem cell maintenance and expression of stem cell characteristics. CDC45 may indirectly regulate the expression of stem cells by interacting with epigenetic modification factors such as histone acetyltransferase (HAT) and deacetylase (HDAC) [49].

To demonstrate a stronger mechanistic link, it is indeed warranted to incorporate overexpression experiments in future studies. Unfortunately, due to resource constraints and technical limitations, we were unable to conduct these experiments in the current study. However, our comprehensive analysis of publicly available databases revealed consistent and significant associations between CDC45 expression and the observed stemness and cell cycle changes. Future research should focus on experimentally validating these findings through robust overexpression studies. Such experiments would provide valuable insights into the functional role of CDC45 in regulating stemness and cell cycle progression.

The precise mechanism by which CDC45 contributes to the observed changes in stemness and cell cycle regulation remains unclear. Our analysis highlights the potential involvement of CDC45 in these processes based on its significant associations with relevant gene expression signatures. However, the exact molecular pathways and downstream effectors through which CDC45 influences stemness and cell cycle control require further exploration. It is plausible that CDC45 may interact with other key regulators or signaling pathways known to modulate stemness and cell cycle progression. Future research should focus on dissecting the intricate interactions between CDC45 and cell cycle checkpoint proteins, such as CHK1 and CHK2, as well as its potential involvement in epigenetic modifications, such as histone acetylation and deacetylation.

In comparison to prior published literature on CDC45 and its role in proliferation and stemness, our study adds novel insights by comprehensively integrating data from various publicly available databases. By employing a systematic analytical approach, we identified significant associations between CDC45 expression and alterations in stemness and cell cycle regulation. This not only reinforces the previously reported role of CDC45 in proliferation and stemness but also expands our understanding by providing a broader molecular context. Furthermore, our study establishes associations with specific gene expression signatures, which may shed light on potential downstream effectors or pathways regulated by CDC45. Although further research is needed to overcome the limitations of our study, the insights gained thus far offer a promising avenue for developing targeted therapeutic strategies. By addressing the mechanistic questions surrounding CDC45's role in stemness and cell cycle regulation, we may potentially refine treatment approaches to more effectively suppress tumor cell proliferation and metastasis.

## Conclusion

CDC45-deficient lung cancer cells exhibited cell cycle arrest, leading to the inhibition of stemness and metastasis. These findings provide important insights into understanding lung cancer metastasis and stemness.

## Supplementary Information

**Additional file 1: Figure S1.** Expression of CDC45 in A549 and H1299. (A) The expression of CDC45 in A549 cells was detected by immunofluorescence. (B) The expression of CDC45 in H1299 cells was detected by immunofluorescence. **Figure S2.** Western blot was used to detect the knock-down of CDC45 in A549 and H1299 cells. (A) CDC45 was knocked down in A549 cells. (B) CDC45 was knocked down in H1299 cells. **Figure S3.** Construction of stable cells by adenovirus transfection. (A) PLVX-shRNA-nc, PLVX-shRNA-CDC45, PMD-2G and PsPax2 were co-transfected into 293T cells with lipo2000, respectively, and the results of 293T plain light and GFP fluorescence were observed 24 hours later; (B) Collect virus supernatant of 293T cells, transfect LA-4 respectively and screen with puromycin to obtain stable cell lines, and the results of plain light and GFP fluorescence of LA-4. **Figure S4.** Statistical chart of immunofluorescence quantitative analysis of tumor tissue in mice. (A) quantitatively analyze the expression of CDC45 in sh_CDC45_NC and sh_CDC45 groups. (B) Quantitatively analyze the expressions of CDC45, E-cadherin and vimentin in sh_CDC45_NC and sh_CDC45 groups. (C) Quantitatively analyze the expression of CDC45 and CDK2 in sh_CDC45_NC and sh_CDC45 groups. (D) Quantitatively analyze the expression of CDC45 and Nanog in sh_CDC45_NC and sh_CDC45 groups.

## Data Availability

The data sets generated and/or analyzed during the present study are available from the corresponding author on reasonable request.
