# Supplementary material for: CDC45 promotes the stemness and metastasis in lung adenocarcinoma by affecting the cell cycle
Source: J Transl Med. 2024 Apr 8;22:335. doi: 10.1186/s12967-024-05038-5 (PMC11000299; doi:10.1186/s12967-024-05038-5)
Supplement: Supplementary file 1 — Additional file 1: Figure S1. Expression of CDC45 in A549 and H1299. (A) The expression of CDC45 in A549 cells was detected by immunofluorescence. (B) The expression of CDC45 in H1299 cells was detected by immunofluorescence. Figure S2. Western blot was used to detect the knock-down of CDC45 in A549 and H1299 cells. (A) CDC45 was knocked down in A549 cells. (B) CDC45 was knocked down in H1299 cells. Figure S3. Construction of stable cells by adenovirus transfection. (A) PLVX-shRNA-nc, PLVX-shRNA-CDC45, PMD-2G and PsPax2 were co-transfected into 293T cells with lipo2000, respectively, and the results of 293T plain light and GFP fluorescence were observed 24 hours later; (B) Collect virus supernatant of 293T cells, transfect LA-4 respectively and screen with puromycin to obtain stable cell lines, and the results of plain light and GFP fluorescence of LA-4. Figure S4. Statistical chart of immunofluorescence quantitative analysis of tumor tissue in mice. (A) quantitatively analyze the expression of CDC45 in sh_CDC45_NC and sh_CDC45 groups. (B) Quantitatively analyze the expressions of CDC45, E-cadherin and vimentin in sh_CDC45_NC and sh_CDC45 groups. (C) Quantitatively analyze the expression of CDC45 and CDK2 in sh_CDC45_NC and sh_CDC45 groups. (D) Quantitatively analyze the expression of CDC45 and Nanog in sh_CDC45_NC and sh_CDC45 groups. [file 12967_2024_5038_MOESM1_ESM.docx]

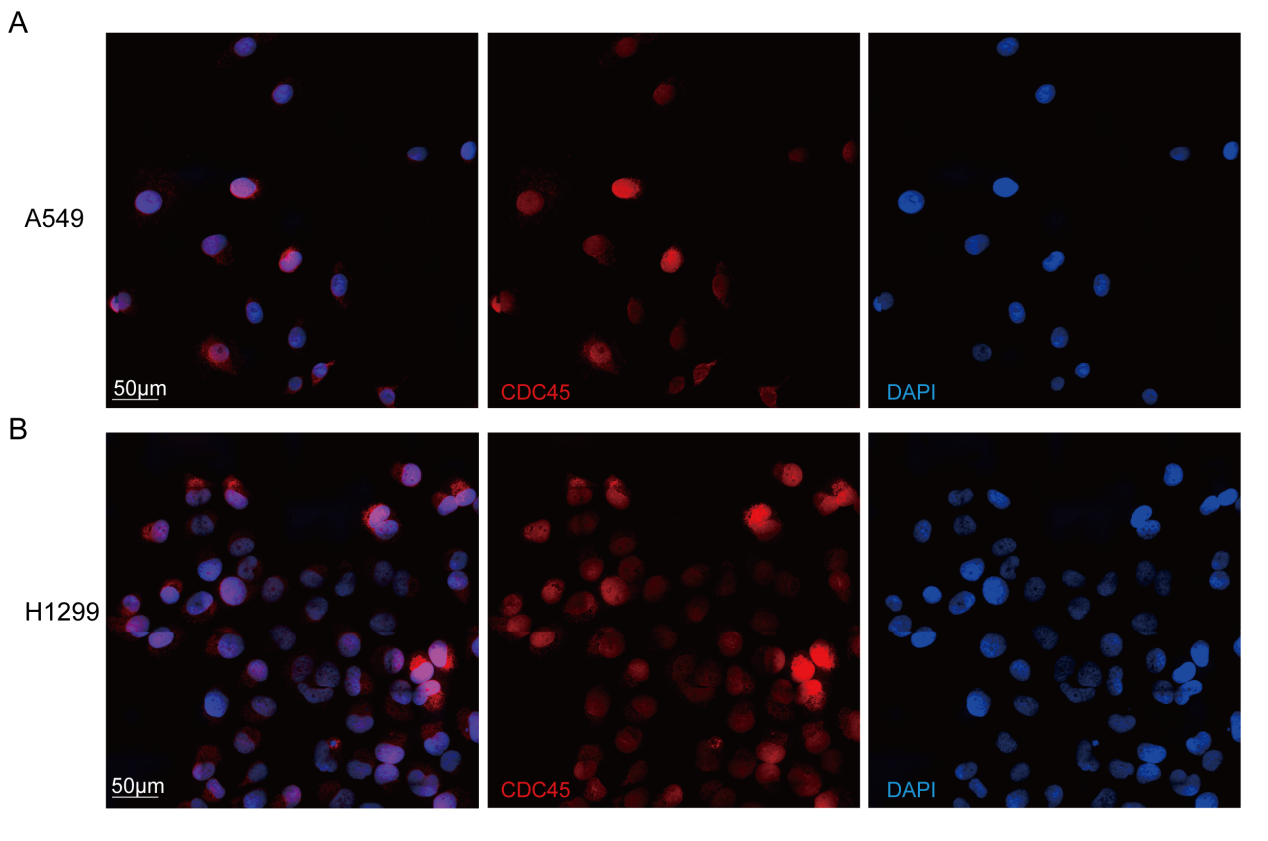


Additional Figure 1

Expression of CDC45 in A549 and H1299. (A) The expression of CDC45 in A549 cells was detected by immunofluorescence. (B) The expression of CDC45 in H1299 cells was detected by immunofluorescence.


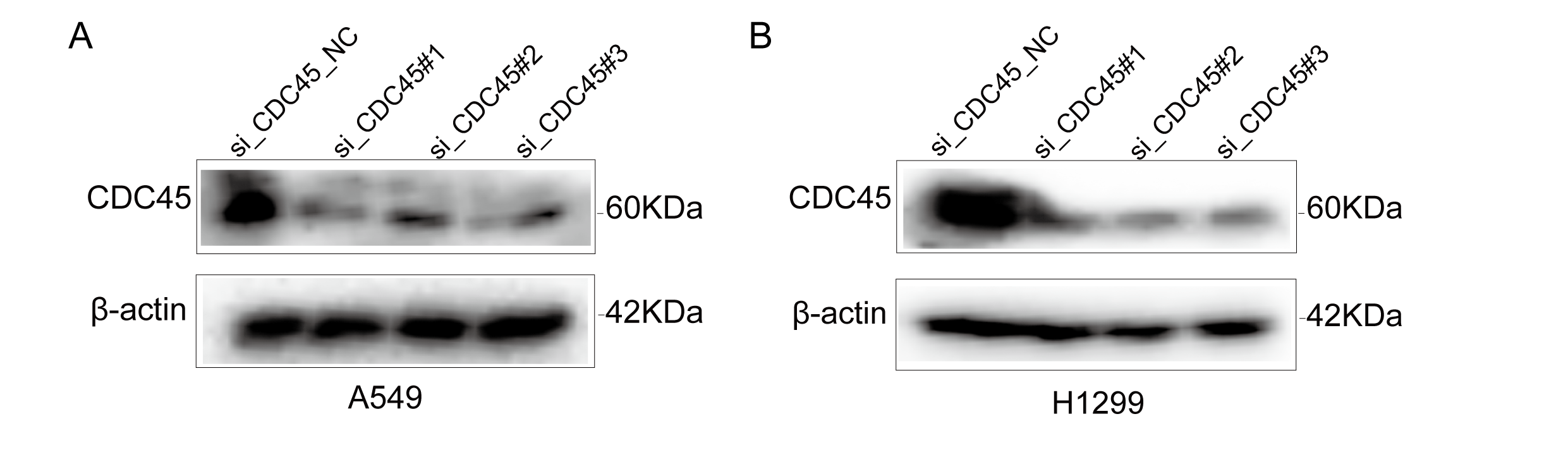


Additional Figure 2

Western blot was used to detect the knock-down of CDC45 in A549 and H1299 cells. (A) CDC45 was knocked down in A549 cells. (B) CDC45 was knocked down in H1299 cells.


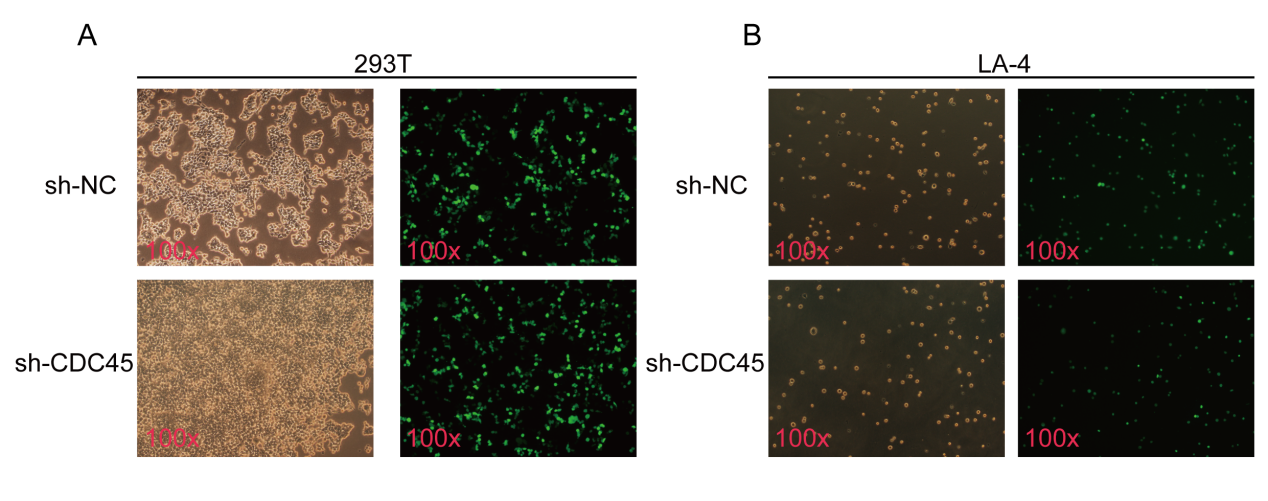


Additional Figure 3

Construction of stable cells by adenovirus transfection. (A) PLVX-shRNA-nc, PLVX-shRNA-CDC45, PMD-2G and PsPax2 were co-transfected into 293T cells with lipo2000, respectively, and the results of 293T plain light and GFP fluorescence were observed 24 hours later; (B) Collect virus supernatant of 293T cells, transfect LA-4 respectively and screen with puromycin to obtain stable cell lines, and the results of plain light and GFP fluorescence of LA-4.

**
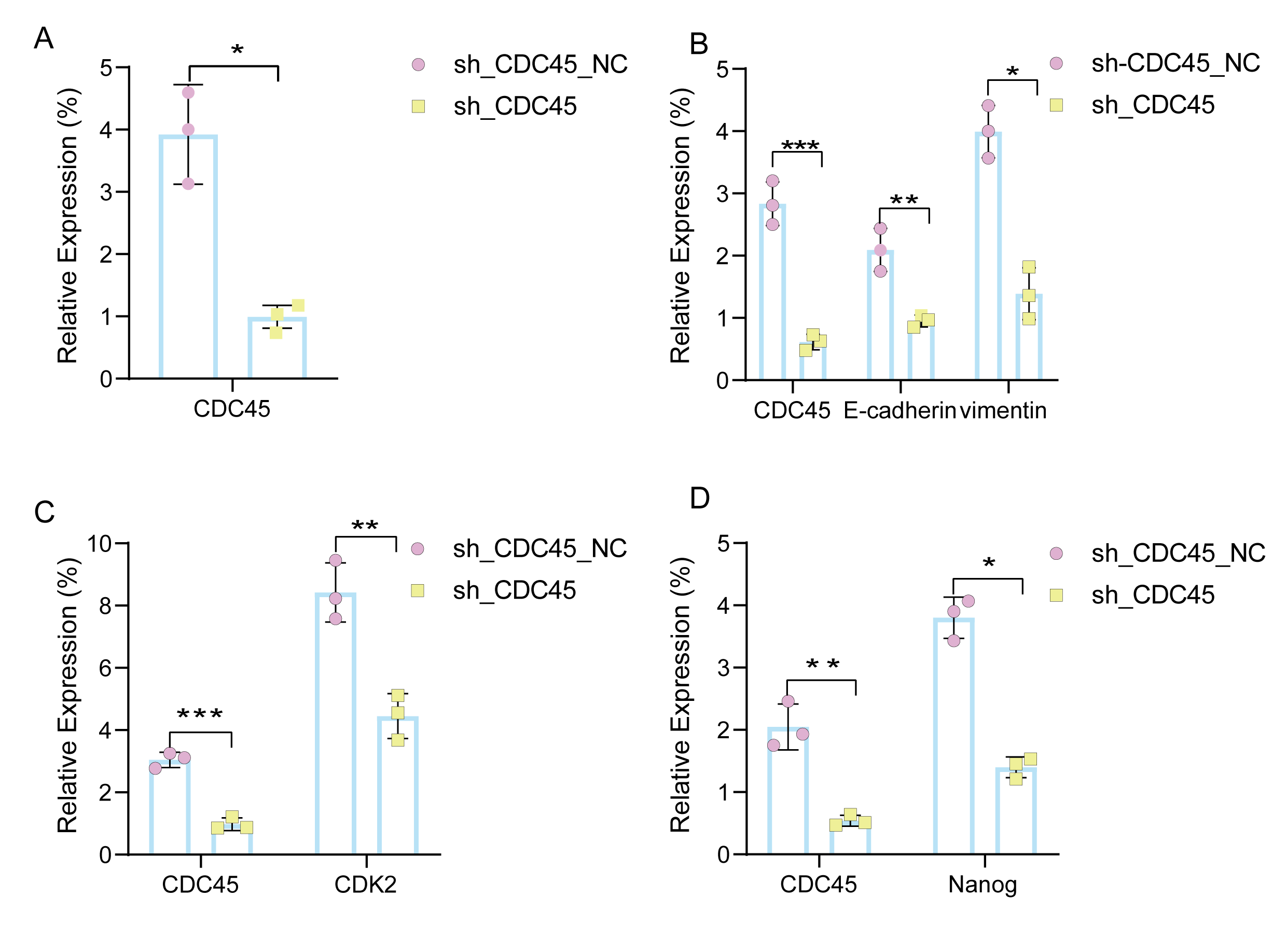
**

Additional Figure 4

Statistical chart of immunofluorescence quantitative analysis of tumor tissue in mice. (A) quantitatively analyze the expression of CDC45 in sh_CDC45_NC and sh_CDC45 groups. (B) Quantitatively analyze the expressions of CDC45, E-cadherin and vimentin in sh_CDC45_NC and sh_CDC45 groups. (C) Quantitatively analyze the expression of CDC45 and CDK2 in sh_CDC45_NC and sh_CDC45 groups. (D) Quantitatively analyze the expression of CDC45 and Nanog in sh_CDC45_NC and sh_CDC45 groups.
